# Dasabuvir suppresses esophageal squamous cell carcinoma growth in vitro and in vivo through targeting ROCK1

**DOI:** 10.1038/s41419-023-05633-2

**Published:** 2023-02-13

**Authors:** Xinning Liu, Yanan Jiang, Hao Zhou, Xiaokun Zhao, Mingzhu Li, Zhuo Bao, Zitong Wang, Chenyang Zhang, Zhenliang Xie, Jimin Zhao, Zigang Dong, Kangdong Liu, Zhiping Guo

**Affiliations:** 1 Department of Pathophysiology, School of Basic Medical Sciences, Zhengzhou University, Zhengzhou, Henan China; 2 China-US Hormel (Henan) Cancer Institute, Zhengzhou, Henan China; 3 Department of Pulmonary and Critical Care Medicine, Huashan Hospital, Fudan University, Shanghai, China; 4 State Key Laboratory of Esophageal Cancer Prevention and Treatment, Zhengzhou University, Zhengzhou, China; 5 Henan Provincial Cooperative Innovation Center for Cancer Chemoprevention, Zhengzhou, China; 6 Research Center of Basic Medicine, Academy of Medical Sciences, Zhengzhou University, Zhengzhou, Henan China; 7 Cancer Chemoprevention International Collaboration Laboratory, Zhengzhou, China; 8 Fuwai Central China Cardiovascular Hospital, Zhengzhou University, Zhengzhou, China

**Keywords:** Cancer prevention, Proteomics, Target identification

## Abstract

Esophageal squamous cell carcinoma (ESCC) is an upper gastrointestinal cancer with high morbidity and mortality. New strategies are urgently needed to prolong patients’ survival. Through screening FDA-approved drugs, we found dasabuvir, a drug approved for hepatitis C virus (HCV) treatment, suppressed ESCC proliferation. Dasabuvir could inhibit the growth of ESCC cells in a time and dose-dependent manner and arrested cell cycle at the G0/G1 phase. The antitumor activity was further validated in vivo using patient-derived xenograft tumor models. In terms of mechanism, we unveil that dasabuvir is a Rho-associated protein kinase 1 (ROCK1) inhibitor. Dasabuvir can bind to ROCK1 and suppress its kinase activity, thus downregulating the phosphorylation of ERK1/2 by ROCK1 and the expression of cyclin-dependent kinase 4 (CDK4) and cyclin D1. These results provide evidence that dasabuvir suppresses ESCC growth in vivo and in vitro through blocking ROCK1/ERK signaling pathway.

## Introduction

Esophageal cancer is an upper-digestive tumor ranks 7th in terms of incidence (604,000 new cases) and 6th in overall mortality (544,000 deaths) worldwide in 2020 [1]. Esophageal squamous cell carcinoma (ESCC) accounts for 90% of esophageal cancer cases in China [2]. Currently, endoscopic treatment, surgery, chemotherapy, radiotherapy, chemoradiotherapy, immunotherapy, targeted therapy and palliative treatment are treatment options for ESCC patients [3, 4]. However, the high recurrent rate and lack of recurrence preventive drugs lead to poor prognosis. Therefore, chemopreventive drugs for ESCC are urgently needed.

Screening drugs approved by the FDA is an effective strategy for finding drugs to prevent the occurrence and recurrence of cancer [5, 6], such as metformin [7], aspirin [8], disulfiram [9] and other drugs exhibit anticancer roles via various signaling pathways. Through such a strategy, we found dasabuvir, an anti-hepatitis C virus (HCV) drug, had an obvious inhibitory effect on ESCC which had not been reported before. Dasabuvir can combine with other direct-acting antiviral drugs to achieve high cure rates in a variety of interferon-free regiments and low adverse reactions as a non-nucleoside NS5B polymerase inhibitor [10–12].

Rho-associated coiled-coil kinases 1 (ROCK1) is a classical serine-threonine kinase which could regulate the cytoskeleton through phosphorylating the downstream substrates and increasing the stability of actin filament and generation of actomyosin contractility [13]. ROCK1 plays an important role in regulating cell movement, angiogenesis and migration which can promote the growth, proliferation, survival of tumor cells by regulating the tumor microenvironment [14]. ROCK1 has been found to be associated with dozens of cancers such as prostate cancer [15–18], laryngeal squamous cell carcinomas [19], nasopharyngeal carcinoma [20], osteosarcoma [21, 22], breast cancer [23], and gastric cancer [24]. It was reported that overexpression of ROCK1 was also significantly associated with the progression of ESCC and predicted poor prognosis [25–27]. These data indicate that ROCK1 is a potential target for ESCC treatment or chemoprevention.

In the current study, we found dasabuvir had cytotoxic effect on ESCC cells, and effectively inhibited the proliferation and anchor-independent and dependent growth of ESCC cells in vitro. Dasabuvir is a ROCK1 inhibitor that can block the ROCK1/ERK signaling pathway, consequently downregulate the expression of CDK4 and cyclin D1, thus arresting ESCC cells in the G0/G1 phase. Importantly, dasabuvir can inhibit the growth of ESCC tumors in vivo. This study provides an experimental basis for future clinical application of dasabuvir for ESCC chemoprevention.

## Materials and methods

### Chemicals

Dasabuvir (PubChem CID: 56640146) was purchased from ATK Chemical Company (Shanghai, China). Dasabuvir sodium tablets (Exviera) used in vivo was purchased from AbbVie Ireland NL B.V (Sligo, Ireland). RPMI-1640 medium and FBS were purchased from Biological Industries. DAPI, PBS, RNase and PI were purchased from Solarbio Science & Technology Co (Beijing, China). Polybrene, DMSO and BME powder were purchased from Sigma-Aldrich (Shanghai) Trading Co, Ltd (Shanghai, China).

### Cell culture

The ESCC cell lines (KYSE150 and KYSE450) were purchased from the Chinese Academy of Sciences Cell Bank (Shanghai, China). These cell lines were authenticated by STR profiling and cultured in RPMI-1640 medium supplemented with 10% FBS and 1% penicillin /streptomycin at 37 °C in a 5% CO_2_ incubator.

### Cell toxicity and proliferation assay

Cell viability and proliferation assays were performed as described previously [28]. The cell number was assessed by DAPI staining after treatment with dasabuvir (0, 2.5, 5, 10, or 15 μM) for 0, 24, 48, 72, or 96 h. The cells were photographed and counted using IN Cell Analyzer 6000 (GE Healthcare, American U.S.) and the knockdown cells were measured by MTT assay.

### Anchorage independent cell growth assay

After 3 mL 0.6% agar medium containing different concentrations of dasabuvir (0, 2.5, 5, 10, or 15 μM) was added into each well of a 6-well plate and solidified, 1 mL 0.3% agar medium containing different concentrations of dasabuvir (0, 2.5, 5, 10, or 15 μM) and 8000 cells was plated over the solidified 0.6% agar medium. After culturing for 7–14 days, the cell clones were photographed and counted using IN Cell Analyzer 6000.

### Anchorage dependent cell growth assay

Each well of 6-well plate was seeded with 200 cells. After culturing with different concentrations of dasabuvir (0, 2.5, 5, 10, or 15 μM) for 7–10 days, the cell clones were fixed, stained and counted.

### Mass spectrometry and omics analysis

KYSE150 cells were treated with 15 μM dasabuvir for 24 h. The cells were then collected and lysed by ultrasonic wave. After centrifugation, the protein concentration was determined. Trypsin enzymatic hydrolysis was performed to obtain polypeptides. After modification and enrichment, the polypeptides were separated and analyzed by mass spectrometry. Maxquant (v1.5.2.8) was used to retrieve secondary mass spectrometry data.

### Protein extraction and Western blotting analysis

KYSE150 and KYSE450 cells were treated with dasabuvir (0, 2.5, 5, 10, or 15 μM) for 24 h. Protein extraction and Western blotting analysis were performed as described previously [29]. The primary antibodies made against ROCK1 (CST: #4035 T), phospho-ERK1/ERK2 (Thr185, Tyr187; Invitrogen: 700012), P44/42 MAPK (ERK1/2; CST: #9102S), Cyclin D1 (Wanlei: WL01435a), and CDK4 (CST: #12790) were used at 1: 1000 dilutions. The protein bands were visualized using a chemiluminescence reagent.

### Kinase prediction, target prediction, and correlation analysis

The kinase prediction of dasabuvir was carried out using iGPS1.0 (http://igps.biocuckoo.org/). The target prediction for dasabuvir was performed using SwissTargetPrediction (http://www.swisstargetprediction.ch/). Correlation analysis of ROCK1 and MAPK1 was performed using the TCGA database (https://www.aclbi.com/static/index.html#/).

### Computational modeling of dasabuvir with ROCK1

The docking of dasabuvir to ROCK1 was performed using the Schrodinger Suite 2016, and the ROCK1 crystal structure (PDB: 2ESM) was downloaded from the PDB (https://www.rcsb.org/).

### Pull-down assay

Preparation of dasabuvir-Sepharose 4B beads was performed as reported [30]. KYSE150, KYSE450, 293 T and 293 F (overexpressing ROCK1) cell lysates (500 μg), recombinant human active ROCK1 (200 ng) were incubated with dasabuvir-Sepharose 4B(100 µL) and Sepharose 4B beads (100 µL) alone (as a control) in reaction buffer. The binding of proteins was verified through Western blotting.

### ATP competition assay

Recombinant human active ROCK1 (100 ng) were incubated with dasabuvir-Sepharose 4B (100 µL) and Sepharose 4B (100 µL) alone (as a control) beads in reaction buffer with different concentration ATP (10 or 100 µM). The binding of proteins was verified through Western blotting.

### In vitro kinase assay

The kinase reaction system was consisted of recombinant human active ROCK1 (30 ng), ERK1 protein (300 ng), ERK2 protein (150 ng), ATP (20 μM) and kinase buffer (25 μL). The total reaction system was placed in a 30 °C water bath for 30 min incubation. The phosphorylation of ERK1 and ERK2 was detected by Western blotting.

### Protein purification

ROCK1 (NM_005406) cDNA clone (number: G124885) was purchased from YouBia Biotechnology Company (Chongqing, China). ROCK1 kinase domain (117-535aa) and the mutated ROCK1 kinase domain (M156A, L202A, and D205A) PCR product were inserted into pGEX-6p-1 vector between SmaI and SalI restriction sites to obtain pGEX-6p-1-ROCK1 and ROCK1 (M156A, L202A, and D205A) plasmids (Supplementary Table S1). These plasmids were transformed into chemically competent E. coli BL21 (DE3) cells. The harvested cells were lysed via sonication and centrifugation. The recombinant ROCK1 protein was purified through a HisTrap column (GE Healthcare) and a HiTrap Q column (GE Healthcare), and then loaded onto a Superdex 200 10/300 gel filtration column.

### Immunofluorescence assay

Cells were incubated overnight at 4 °C with primary antibodies containing ROCK1 (SCBT: sc-17794) and p-MAPK1, followed by secondary antibodies containing FITC (Abbkine: A22120) and TRTIC (GeneTex: GTX26744) for 2 h. After DAPI staining, the images were captured by IN Cell Analyzer 6000 and analyzed by Image J.

### Gene set enrichment analysis

GSEA V4.1.0 software package was used to analyze the differences in protein expression between the treatment group and the control group in phosphoproteomics and proteomics data.

### Cell cycle assay

Cells were plated into 60 mm culture dishes (2 × 10^5^ cells/dish). The cells were starved for 24 h and treated with dasabuvir. Cells were fixed in 1 mL of cold 70% ethanol and stored at −20 °C for 24 h. After treated with RNase (100 mg/mL) and stained with PI (20 mg/mL). Cells were then analyzed by Flow Cytometer (BD Biosciences, San Jose, CA).

### Generation of stable ROCK1 knock-down cell lines

The ROCK1 shRNA sequences (Supplementary Table S2) were designed using the siRNAext program (http://jura.wi.mit.edu siRNAext). These shROCK1 plasmids were transferred to 293 T cells to collect the shRNA lentiviral particles. KYSE150 and KYSE450 cells (60% confluent) were cultured with DMEM containing 8 μg/mL polybrene and shRNA lentiviral particles. A medium containing puromycin (KYSE150 2 μg/mL, KYSE450 1 μg/mL) was used to select shROCK1 cells.

### Patient-derived xenograft mouse model

Female SCID mice (5–6 weeks) were purchased from Vital River (Beijing, China). The study protocol was approved by the Animal Care and Use Committee of Zhengzhou University (Zhengzhou, Henan Province, China). The samples of ESCC tumor tissue were obtained from the Linzhou Tumor Hospital. All patients provided written informed consent to use the tissue samples. EG20 was from a male, 46 years old, with T2N0M0II, with moderately differentiated medullary squamous cell carcinoma. LEG110 was from a male, 69 years old, T3N1M0IIIb, with moderately differentiated medullary squamous cell carcinoma. LEG34 was from a female, 68 years old, T4N0M0III, with moderately differentiated medullary squamous cell carcinoma. The process of building the PDX mouse model has been described previously [31]. When the average tumor volume reached 100 mm^3^, mice were randomly divided into 3 groups: gavage with solvent control (0.9% saline), 10 mg/kg and 50 mg/kg made dasabuvir until the average tumor volume of the control group reached 1000 mm^3^. The weight of the mice was monitored every 2 days, and tumor volume was measured every 3 days. Tumor volume was calculated using the following formula: tumor volume = length × width ^2^/2.

### HE staining and immunohistochemical

5 μm paraffin organ tissue sections were prepared, dewaxed, stained by hematoxylin and eosin (HE). 5 μm paraffin tumor tissue sections were prepared, dewaxed, exposed to antigen, and incubated overnight with primary antibody (1: 50) at 4 °C. The secondary antibody was incubated at 37 °C for 30 min, then stained with DAB and restained with hematoxylin. After dehydration, slides were installed, scanned by tissue fax (version 4.2) and analyzed by the Image Pro Plus software program (Media Cybernetics, Rockville, MD).

### Statistical analysis

On the premise of homogeneity of variance, the difference between the two groups was calculated by a one-way ANOVA test (SPSS 20.0). All experiments were performed at least three times, and *p* < 0.05 was considered significant.

## Results

### Dasabuvir inhibits ESCC cell proliferation in vitro

**Fig. 1:**
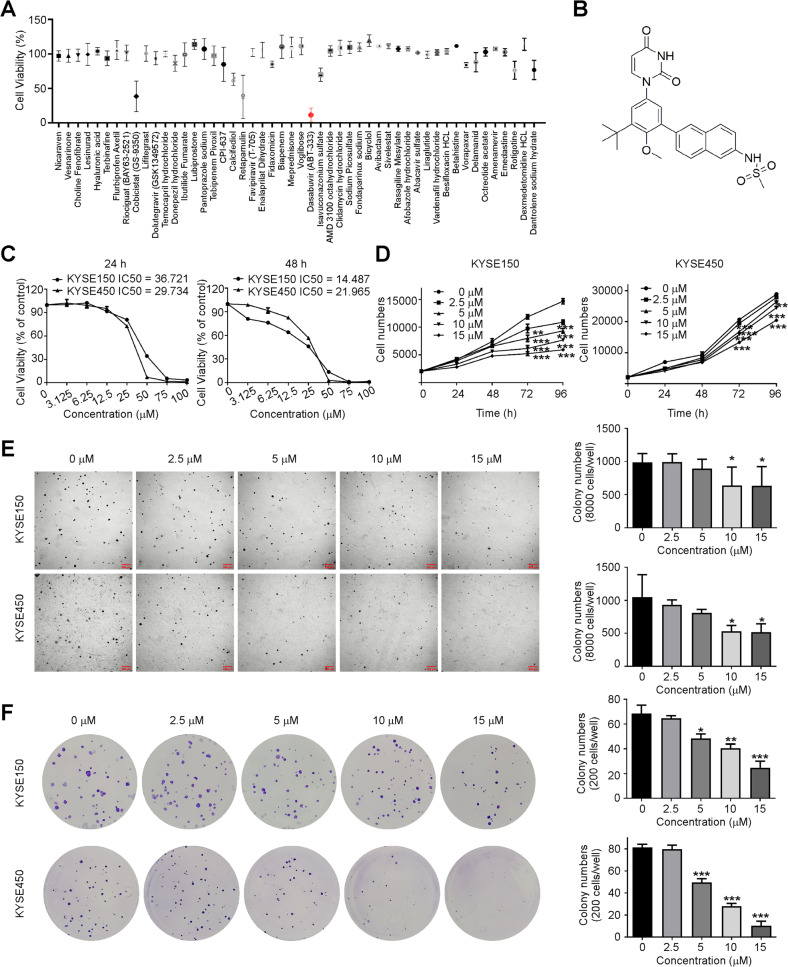
Dasabuvir inhibits ESCC cell proliferation in vitro. **A** KYSE450 cells were used to screen the cytotoxicity of 50 drugs (*n* = 3). The concentration of each drug was 50 μM. **B** Chemical structure of dasabuvir. **C** The effect of dasabuvir on the cell viability of KYSE150, KYSE450 cells. Cell viability was detected by DAPI staining (*n* = 3). **D** The effect of dasabuvir on the proliferation of KYSE150, KYSE450 cells. Proliferation ability was detected by DAPI staining (*n* = 3). **E** Representative images of anchorage independent cell growth assay (left) and quantitative analyses of colony numbers (right, *n* = 3). **F** Representative images of anchorage dependent cell growth assay (left) and quantitative analyses of colony numbers (right, *n* = 3). Data were analyzed by one-way ANOVA test and the asterisk indicated a significant (*p < 0.05, **p < 0.01 and ***p < 0.001) difference compared with the control group.

To find a drug can inhibit ESCC, we screened FDA-approved drugs (Fig. 1A, Supplementary Fig. S1) with cytotoxicity assays and found dasabuvir (Fig. 1B) showed obvious cytotoxicity on KYSE450 cells. To further assess its inhibitory effects, we treated the human ESCC cell lines KYSE150 and KYSE450 with various concentrations (0, 2.5, 5, 10, or 15 μM) of dasabuvir according to the IC50 of these two cell lines (Fig. 1C). And the IC50 values of KYSE150 and KYSE450 cells were 36.72 and 29.73 μM at 24 h, and 14.49 and 21.97 μM at 48 h, respectively. The results indicated that dasabuvir markedly suppressed cell growth in a dose-dependent manner (Fig. 1D). In the anchorage independent (Fig. 1E) and anchorage dependent (Fig. 1F) ESCC cell growth assays, dasabuvir showed strong dose-dependent growth inhibition. These data indicated that dasabuvir effectively inhibited ESCC proliferation in vitro.

### Dasabuvir suppresses ROCK1/ERK signaling pathway

To investigate the underlying molecular mechanism of dasabuvir on ESCC, phosphoproteomics were performed after KYSE150 cells were treated with dasabuvir for 24 h. Compared with the control group (DMSO), 8387 phosphorylation sites of 2952 proteins contained quantitative information were identified. To ensure that the results were reliable, the identification data was filtered by a localization probability >0.75. Then the filtered protein quantification group was normalized to remove the influence of protein expression on the modified signal and used for subsequent bioinformatics analysis (Supplementary Fig. S2A and S2B).

**Fig. 2:**
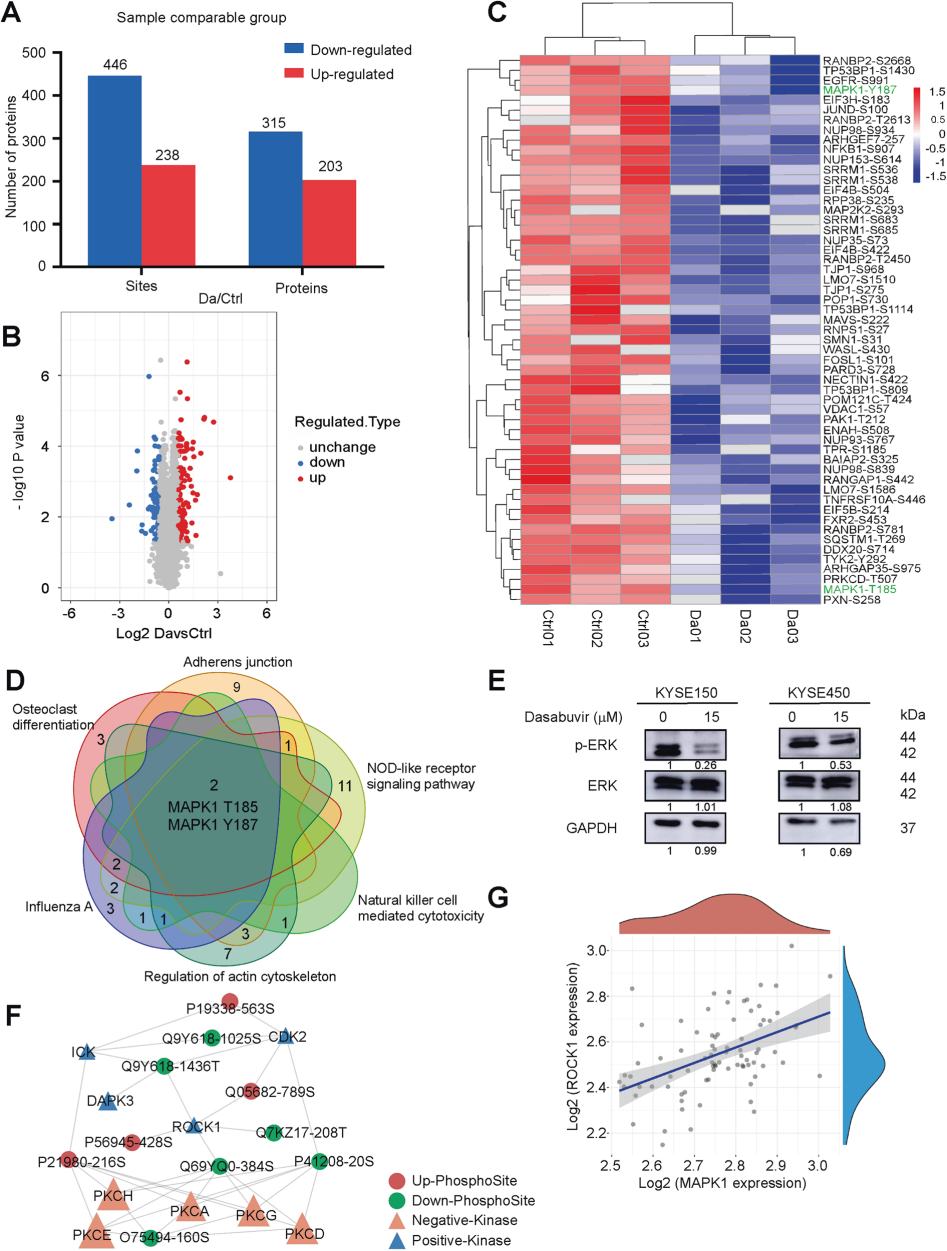
Dasabuvir acts through ROCK1/ERK signaling pathway. **A** Histogram of the number of changed protein and phosphorylation sites between control (DMSO) and dasabuvir (15 μM) treated groups. **B** Volcano plot of changed phosphorylation sites between control (DMSO) and dasabuvir (15 μM) treated groups. **C** Heat map of significantly downregulated phosphorylation sites. **D** Venn diagram showed MAPK1 T185, Y187 were enriched from six KEGG pathways. **E** The selected target was validated by Western blotting. Representative images are shown. **F** A kinase regulatory network centered on ROCK1 mapped by Cytoscape. **G** TCGA data showed a positive correlation between ROCK1 and MAPK1 in ESCC (n_pairs_ = 82, *p* = 6.43 × 10^−6^, ρ_Spearman_ = 0.48).

The different sites were screened for a 1.5-fold change threshold and a *t*-test *p*-value < 0.05. Based on these data and criteria, the modification levels of 238 sites in the dasabuvir treatment group were found to be upregulated, and 446 sites were downregulated (Fig. 2A and B). Then we enriched the downregulated phosphorylation sites (Fig. 2C) which belonged to eight signaling pathways (Supplementary Fig. S2C). Interestingly, MAPK1 T185 and Y187 were enriched in 6 of the 8 downregulated pathways (Fig. 2E, Supplementary Fig. S2D). Western blotting results also confirmed the downregulation of p-ERK1/2 (Fig. 2F).

Then SwissTargetPrediction was used to predict the possible targets of dasabuvir. The results showed that ROCK1 ranked first among all 100 predicted targets (Supplementary Table S3). We also performed kinase activity analysis based on mass spectrometry results, as the phosphorylation level may reflect the regulatory state of kinases. A kinase regulatory network was constructed to observe the regulatory relationship between kinases and substrates. ROCK1 was enriched and its kinase activity was inhibited (Fig. 2F). These indicated that ROCK1 was a potential target of dasabuvir. The TCGA data showed that the levels of ROCK1 and MAPK1 were positively correlated in ESCC (Fig. 2G). Consequently, dasabuvir might inhibit cell proliferation via ROCK1/ERK signaling pathway in ESCC.

### Dasabuvir binds to ROCK1 and inhibits its activity

**Fig. 3:**
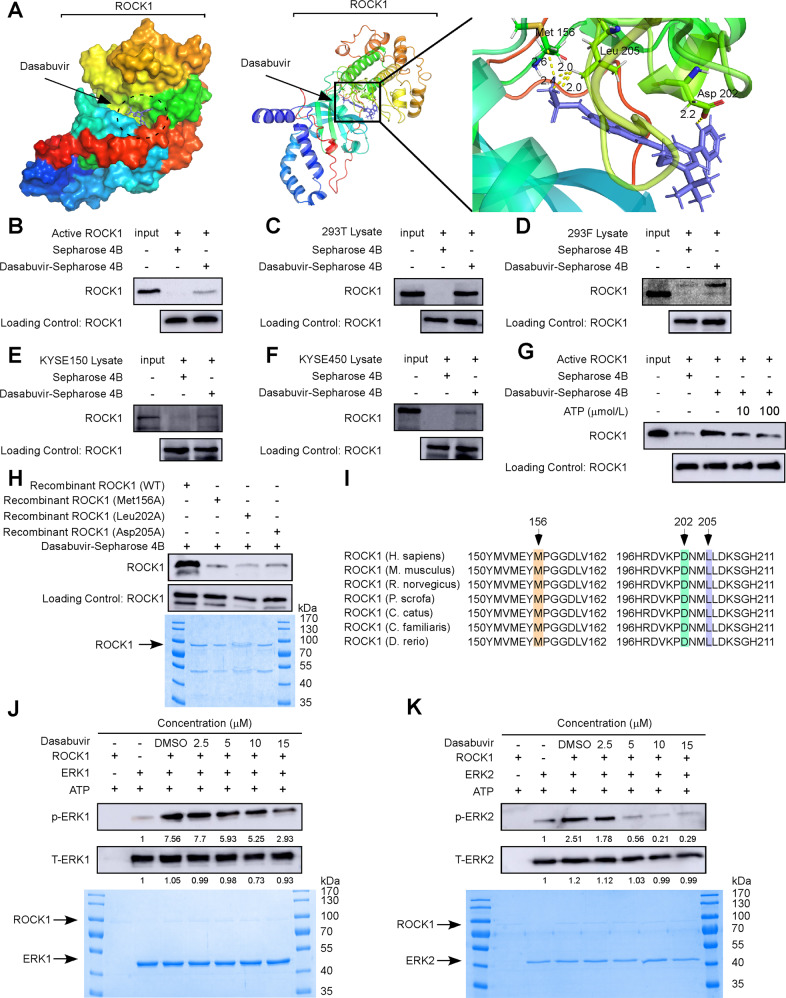
Dasabuvir binds to and inhibits ROCK1 activity. **A** Molecular docking showed dasabuvir could bind to ROCK1 kinase domain. (PDB database: 2ESM) **B** Pull-down assay indicated dasabuvir could bind to recombinant ROCK1 protein. **C**, **D** Pull-down assays indicated dasabuvir could bind to the full-length ROCK1. **E**, **F** Pull-down assays indicated dasabuvir could bind to endogenous ROCK1. **G** ATP competition assay indicated dasabuvir bound to ROCK1 in an ATP competitive way. **H** Dasabuvir bound to WT ROCK1, but not mutant ROCK1 (Met 156, Leu 202, Asp 205) validated by the pull-down assay (upper panel). The SDS-PAGE gel showed the presence of wild type and mutant type ROCK1 proteins (lower panel). **I** Comparison diagram of ROCK1 sequence of different species. **J** In vitro kinase assay showed ROCK1 can activate ERK1, while dasabuvir can inhibit its kinase activity (upper panel). The SDS-PAGE gel showed the presence of ROCK1 and ERK1 (lower panel). **K** In vitro kinase assay showed ROCK1 can activate ERK2, while dasabuvir can inhibit its kinase activity (upper panel). The SDS-PAGE gel showed the presence of ROCK1 and ERK2 (lower panel).

To determine whether ROCK1 is a direct target of dasabuvir or not, we performed molecular docking between dasabuvir and ROCK1. The docking results indicated that dasabuvir bound to ROCK1 at Met 156, Leu 202, and Asp 205 (Fig. 3A), and that these binding sites belong to the protein kinase domain of ROCK1. To verify this binding model, we conjugated dasabuvir with Sepharose 4B beads and conducted pull-down assays. The active ROCK1 kinase domain bound to Sepharose 4B beads conjugated with dasabuvir, but not to Sepharose 4B beads alone (Fig. 3B). To confirm whether dasabuvir binds to full-length ROCK1, we used exogenous and endogenous ROCK1 to perform pull-down assays. The results also showed that dasabuvir directly bound to exogenous full-length ROCK1 protein in 293 T and 293 F cells transfected with pcDNA3.1-ROCK1-HA (Fig. 3C, D) and endogenous ROCK1 in KYSE150 and KYSE450 cells (Fig. 3E, F). In addition, the binding of dasabuvir to ROCK1 was obviously attenuated by the presence of ATP (Fig. 3G).

MEK1/2, as a direct upstream of ERK1/2, was also tested for binding to dasabuvir. The results indicated that dasabuvir did not bind to MEK1/2 (Supplementary Fig. S3A and S3B). In order to further verify the binding sites of ROCK1 to dasabuvir, we mutated Met 156, Leu 202, and Asp 205 to alanine, and purified the wild-type recombinant ROCK1 kinase domain protein and mutated ROCK1 protein. Dasabuvir bound to the wild-type ROCK1 protein but not the mutant protein (Fig. 3H). Comparison of ROCK1 sequences from different species also indicated that these binding sites of ROCK1 sequence were highly conserved (Fig. 3I). And it may be important for dasabuvir to play a role in ROCK1. These results suggested that dasabuvir bound to ROCK1 through the Met 156, Leu 202, and Asp 205 sites in an ATP-competing manner.

An in vitro kinase assay was conducted to examine whether ROCK1 activity can be inhibited by dasabuvir. Activated recombinant ROCK1 kinase protein was mixed with human recombinant ERK1 and ERK2 in the presence of various concentrations of dasabuvir to assess phosphorylation of ERK1 and ERK2. The results showed that phosphorylation of ERK1 and ERK2 were markedly reduced (Fig. 3J, K), indicating that dasabuvir could inhibit ROCK1 kinase activity in a dose-dependent manner. Taken together, these results demonstrated that dasabuvir was an ATP-competed inhibitor of ROCK1.

### Dasabuvir induces G0-G1 cell cycle arrest of KYSE150 and KYSE450 cells through ROCK1/ERK signaling pathway

**Fig. 4:**
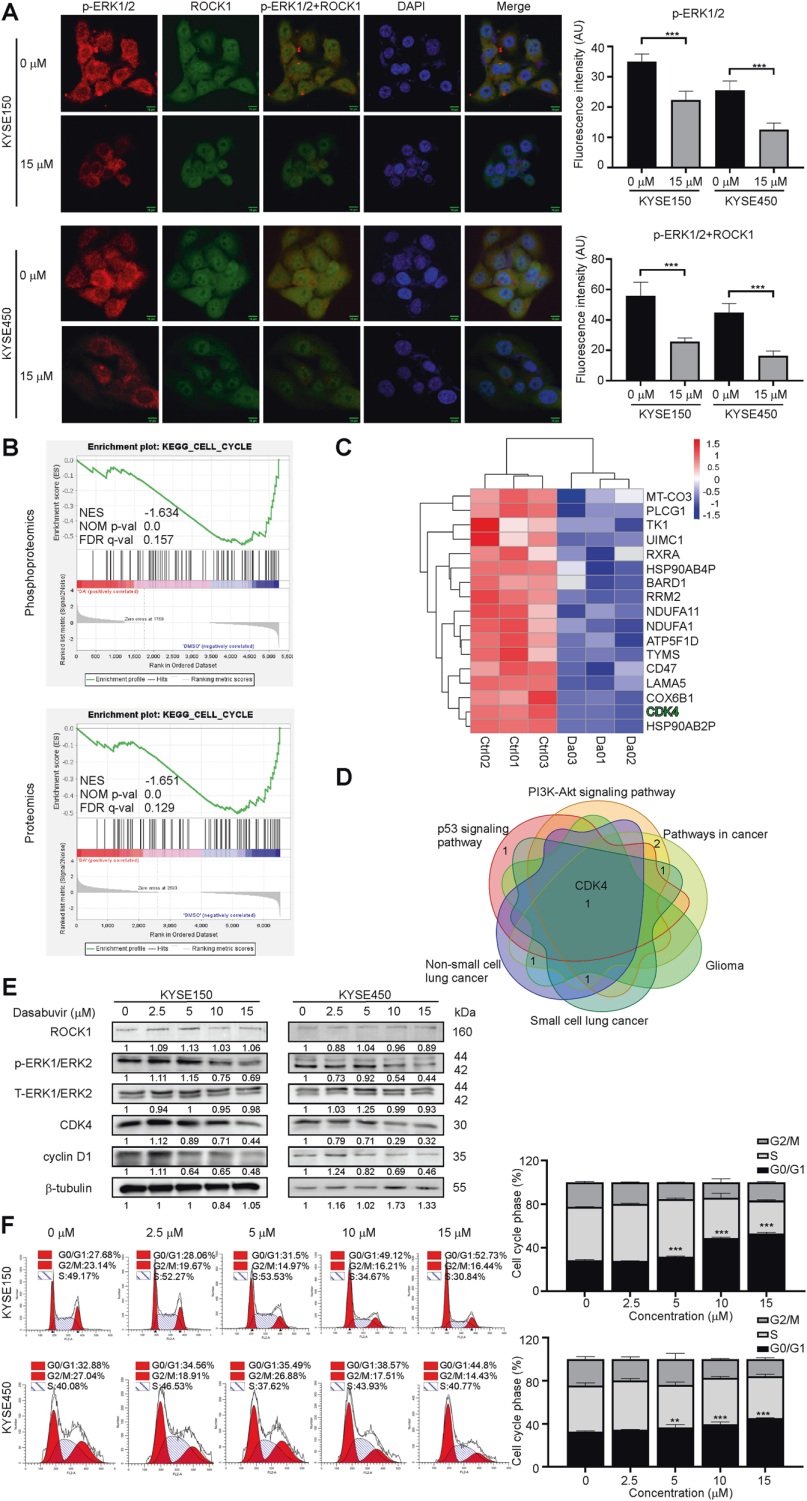
Dasabuvir induces cell cycle arrest through ROCK1/ERK signaling pathway. **A** Immunofluorescence of p-ERK1/2 and ROCK1 in KYSE150 and KYSE450 cells treated with dasabuvir (15 μM) for 24 h were photoed by scanning confocal microscopy. Representative images (left) and quantitative analyses of fluorescence intensity (right, *n* = 5). **B** GSEA Enrichment plot (score curves) from phosphoproteomics (upper) and proteomics (lower). **C** Heat map of significantly downregulated protein sites in KYSE150. **D** Venn diagram showed CDK4 was enriched from the KEGGm pathways. **E** Western blotting showed dasabuvir inhibited the protein levels of p-ERK1/2, CDK4 and Cyclin D1 in a concentration gradient. **F** Cell cycle was analyzed by PI staining and the number of cells in each phase was analyzed by Modfit (*n* = 3). Data were analyzed by one-way ANOVA test and the asterisk indicated a significant (*p < 0.05, **p < 0.01 and ***p < 0.001) difference compared with the control group.

Immunofluorescence results showed that ROCK1 and p-ERK1/2 overlapped in untreated ESCC cells. Dasabuvir treatment blocked the co-localization of the two molecules, and the fluorescence overlap between them became weak (Fig. 4A). These data indicated that dasabuvir blocked ROCK1/ERK signaling pathway in ESCC cells. To further investigate how dasabuvir affects the progression of ESCC through the ROCK1/ERK signaling pathway, GSEA enrichment was performed on the phosphoproteomics and proteomics data. The GSEA results showed that dasabuvir affected the cell cycle signaling pathway of ESCC (Fig. 4B). ROCK1 can regulate the transcription and expression of G1 checkpoint proteins like cyclin D1, CDK2 and CDK4, consequently enhance cell proliferation by promoting cell cycle transition from G0/G1 phase to S phase [32]. The significantly downregulated proteomic protein sites were enriched, the corresponding pathways were located and intersected, and CDK4 was identified (Fig. 4C, D; Supplementary Fig. S4A and S4B). Western blotting analysis indicated that dasabuvir downregulated the levels of p-ERK1/2, cyclin D1, and CDK4 in a dose-dependent manner (Fig. 4E). Cell cycle experiments results showed that dasabuvir significantly blocked the cell cycle at the G0/G1 phase (Fig. 4F), which was consistent with the downregulation of CDK4 and cyclin D1 expression.

### Knockdown of ROCK1 inhibits ESCC cells proliferation and weakens the inhibitory effect of dasabuvir

**Fig. 5:**
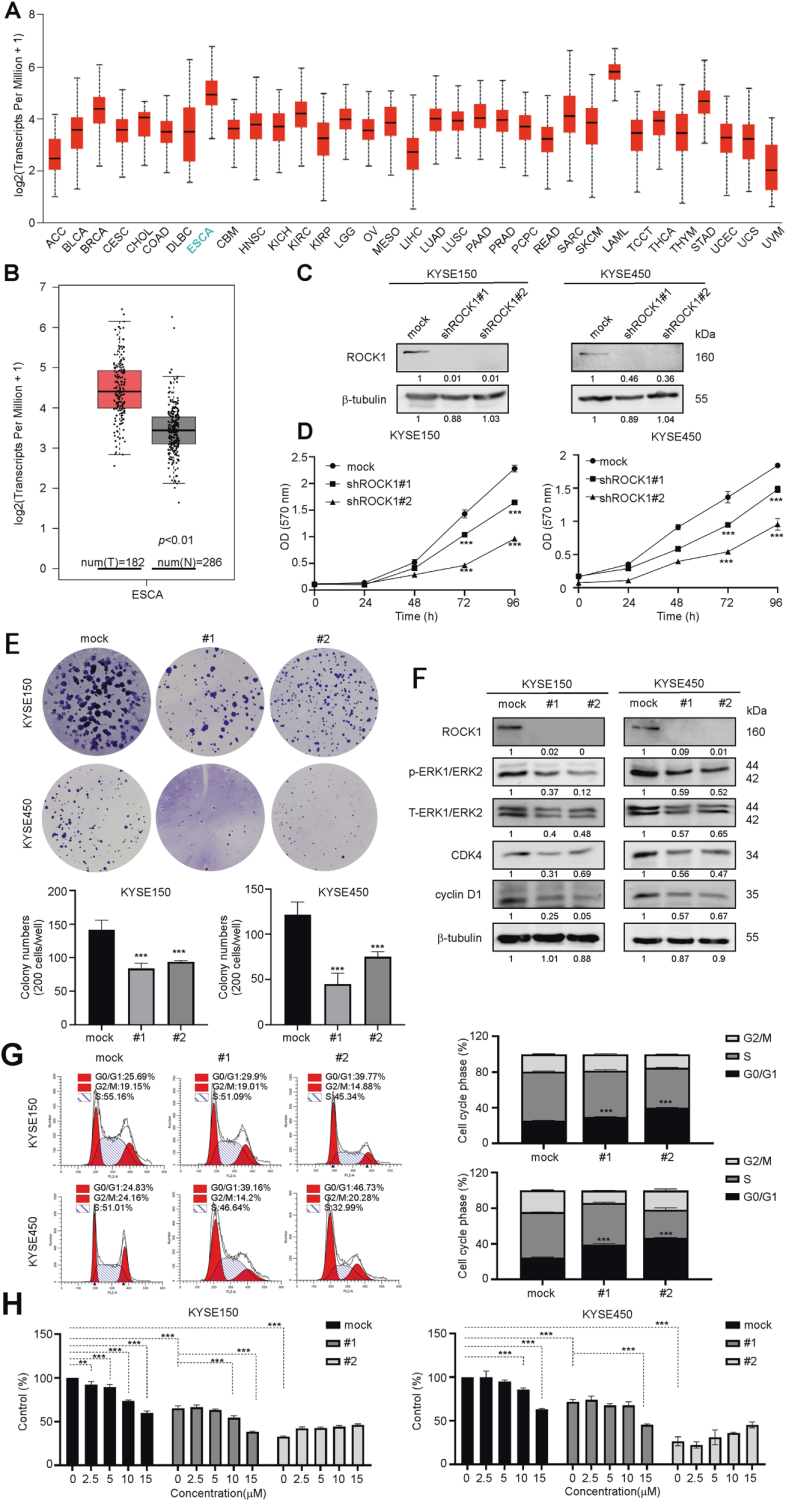
Dasabuvir exerts cancer cell inhibition through ROCK1. **A** The mRNA levels of ROCK1 across different tumors from UALCAN. **B** The mRNA levels of ROCK1 between ESCA and normal tissues from GEPIA. **C** The protein levels of ROCK1 in KYSE150 and KYSE450 cells transfected with sh-Mock or shRNA-ROCK1 was measured by Western blotting. **D** Cell growth was estimated by MTT assay after knocking down ROCK1 (*n* = 3). **E** Representative images of anchorage-dependent cell growth assay (upper) after ROCK1 knocking down and quantitative analyses of colony numbers (lower) (right, *n* = 3). **F** The protein levels of the corresponding downstream targets of after ROCK1 knocking down in KYSE150 and KYSE450 cells were measured by Western blotting. **G** Cell cycle after knocking down ROCK1 was stained with PI and the number of cells in each phase was analyzed by Modfit (*n* = 3). **H** The inhibitory effect of dasabuvir on shROCK1 cells was detected by MTT assay at 72 h (*n* = 3). Data were analyzed by one-way ANOVA test and the asterisk indicated a significant (*p < 0.05, **p < 0.01 and ***p < 0.001) difference compared with the control group.

Based on the data of TCGA and GEPIA, the mRNA levels of ROCK1 in esophageal cancer were higher than that in most cancers (Fig. 5A) and the mRNA levels of ROCK1 in esophageal cancer were higher than that in normal tissue (Fig. 5B). To further verify the function of ROCK1 in ESCC proliferation, we knocked ROCK1 down in KYSE150 and KYSE450 cell lines (Fig. 5C). Then we conducted a proliferation assay and an anchorage dependent cell growth assay. Compared with mock-transfected cells, knockdown of ROCK1 in ESCC cells resulted in suppression of cell proliferation and anchorage-dependent cell growth (Fig. 5D and E). Furthermore, downregulated ROCK1 also inhibited ERK-CDK4/Cyclin D1 signaling pathway (Fig. 5F). With the inactivation of ERK-CDK4/Cyclin D1 signaling pathway, the cell cycle of KYSE150 and KYSE450 cells was also arrested at the G0/G1 phase (Fig. 5G). Compared with mock cells, we also found that the inhibitory effect of dasabuvir was attenuated on ROCK1 knockdown cells (Fig. 5H). These data also indicated that dasabuvir exerted cancer cell inhibitory effect through ROCK1.

### Dasabuvir reduces ESCC PDX tumor growth in vivo

**Fig. 6:**
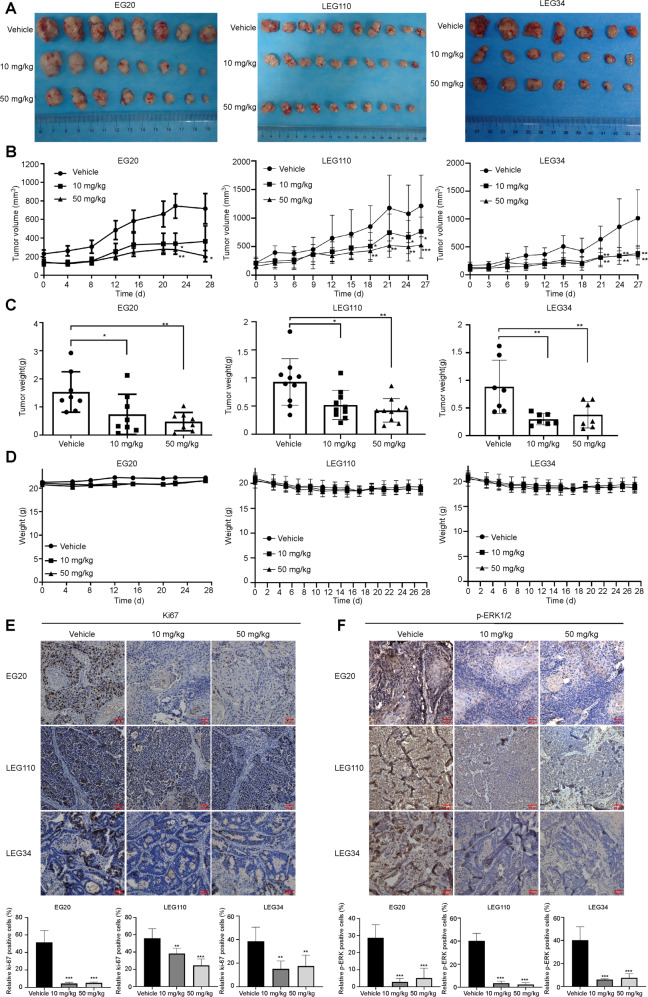
Dasabuvir attenuates ESCC PDX tumors growth in vivo. **A** Photographs showed tumors after treated with the vehicle and dasabuvir for EG20 (*n* = 8), LEG110 (*n* = 10), LEG34 (*n* = 7). **B** Tumor growth curves after treated with the vehicle and dasabuvir for EG20 (*n* = 8), LEG110 (*n* = 10), LEG34 (*n* = 7). **C** Histograms showed tumor weight after treated with the vehicle and dasabuvir for EG20 (*n* = 8), LEG110 (*n* = 10), LEG34 (*n* = 7). **D** Line charts of weight change for EG20 (*n* = 8), LEG110 (*n* = 10), LEG34 (*n* = 7). **E** Ki67 protein levels in ESCC PDX samples were examined by immunohistochemistry (IHC) for EG20 (*n* = 6), LEG110 (*n* = 5), LEG34 (*n* = 6). **F** p-ERK1/2 protein levels were examined by IHC for EG20 (*n* = 5), LEG110 (*n* = 5), LEG34 (*n* = 5). Scale bar = 50 μm. Data were analyzed by one-way ANOVA test and the asterisk indicated a significant (*p < 0.05, **p < 0.01 and ***p < 0.001) difference compared with the control group.

In order to verify the inhibitory effect of dasabuvir in vivo, we chose three ESCC PDX models (EG20, LEG34, LEG110) to conduct further studies. After SCID mice were implanted with tumor fraction, physiological saline or dasabuvir (10 mg/kg and 50 mg/kg) was administered via gavage once daily. The results indicated dasabuvir effectively inhibited tumor growth and tumor volume compared with the vehicle group (Fig. 6A–C, Supplementary Fig. S5A–S5C). Interestingly, no significant body weight changed (Fig. 6D), ruffled fur changed and behavior changed in the mice treated with the drug compared to the vehicle group. There were no pathologic changes in heart, liver, spleen, lung, kidney via HE staining analysis (Supplementary Fig. S6). In addition, the immunohistochemical analysis results showed that the expression of Ki67 and p-ERK1/2 were strongly suppressed in the dasabuvir treatment group compared with the vehicle group (Fig. 6E and F). These results indicated that dasabuvir can inhibit ESCC in vivo.

## Discussion

The high recurrence rate and poor survival in patients with ESCC make the ongoing investigation of ESCC treatment and chemoprevention particularly important. FDA-approved drugs have detailed pharmacokinetic and safety data, making them a good choice for finding drugs for cancer chemoprevention. Through screening FDA-approved drugs (Fig. 1A and Supplementary Fig. S1), we found dasabuvir, an anti-hepatitis C virus drug, had an inhibitory effect on ESCC. Dasabuvir inhibited the growth and colony formation of KYSE150 and KYSE450 (Fig. 1D–F). More importantly, dasabuvir inhibited the growth of PDX tumors in vivo. The dosage 10 mg/kg and 50 mg/kg once daily showed strong inhibitory effects on ESCC, which is lower than the clinical recommended dose (500 mg/60 kg/d for human ≈ 102.5 mg/kg/d for mouse), implying dasabuvir may be an effective drug for ESCC (Fig. 6B–D).

As an anti-HCV drug, dasabuvir interacted with HCV NS5B palm domain to inhibit the encoding of RNA dependent RNA polymerase (RdRp) necessary for replication of HCV genome [33]. Besides, dasabuvir showed antiviral activities against a variety of flaviviruses in vitro [34], dasabuvir partially inhibited Middle East respiratory syndrome coronavirus (MERS-CoV) RdRp activity [35] and inhibited severe acute respiratory syndrome coronavirus 2 (SARS-CoV-2), human rotavirus A (RVA) and human norovirus (HuNoVs) infection [36]. However, the molecular target in eukaryocyte and related function has not been investigated.

ROCK1 was reported to be involved in the progression of several cancers, including ESCC [25–27]. Furthermore, increased ROCK1 mRNA or ROCK1 protein can accelerate disease progression and affect the prognosis and the patient survival [37]. ROCK1 played an important role in the regulation of cell cycle progression. ROCK promoted cell cycle transition from G1 phase to S phase by upregulating cyclin A/D1/D3, CDK2/4/6 and downregulating cell cycle inhibitors CDKN1A, CDKN1B, CDKN2A, CDKN2C, CDKN2D and CDKN4B [38]. ROCK activated the RAS/MAPK pathway and promoted cyclin D1 expression [39]. In this study, we give the first evidence that dasabuvir is a ROCK1 inhibitor. Through phosphoproteomics data, we found MAPK1 T185, Y187 maybe key regulatory sites for dasabuvir on ESCC (Fig. 2E). As well as correlation analysis showing ROCK1 and MAPK1 positively correlated in ESCC (Fig. 2I), further evidences including target prediction, kinase activity analysis, molecular docking and pull-down assays suggested that dasabuvir bound to and inhibited ROCK1 activity (Fig. 2G–H, Fig. 3). The results of GSEA enrichment in this study demonstrated that dasabuvir did affect the cell cycle signaling pathway in ESCC (Fig. 4B) and CDK4 was enriched from proteomics (Fig. 4C–E), Western blotting showed that dasabuvir suppressed the protein levels of CDK4 and cyclin D1 (Fig. 4F) and cell cycle assay also indicated ESCC cells were blocked in the G0/G1 phase (Fig. 4G). Furthermore, knocking down ROCK1 in ESCC cells reduced the inhibitory efficiency of dasabuvir (Fig. 5H). These evidence indicated dasabuvir exert anti-ESCC effect by targeting ROCK1 and its related signaling pathways.

Currently, various ROCK1 inhibitors are used to reduce progression, metastasis and migration of a variety of cancers [37]. Hundreds of ROCK1 inhibitors have been identified and have various therapeutic potentials. ROCK1/2 inhibitors such as fasudil [40, 41], AT13148 [42–44], Y-27632 [45, 46], YM529/ONO-5920 [47, 48], PT-262 [49], WF-536 [50–52] and RKI-1447 [53] have shown significant cancer inhibition [13]. Comparison with other ROCK1 inhibitors, dasabuvir offered more clinical possibilities with more available clinical safety data, which means we can conduct clinical trials to test the inhibitory effect of dasabuvir on ESCC especially patients who have HCV infection.

**Fig. 7:**
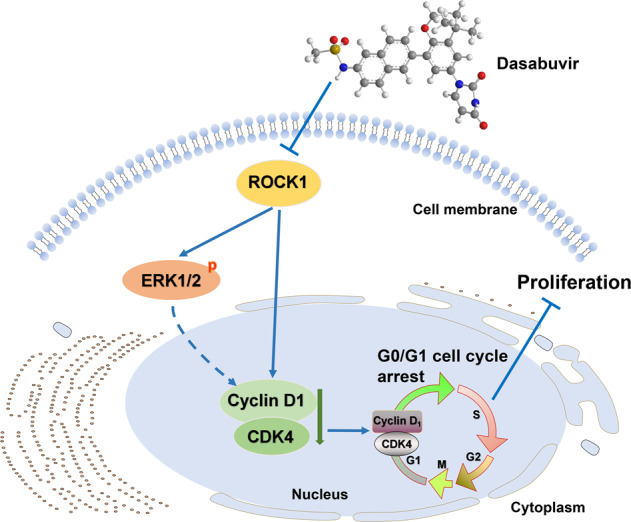
Mechanism diagram of dasabuvir inhibiting esophageal squamous cell carcinoma. Dasabuvir binds to ROCK1 and inhibits its kinase activity, which results in downregulating the phosphorylation of ERK1/2 by ROCK1 and the expression of CDK4 and cyclin D1. Dasabuvir arrests cell cycle at the G0/G1 phase and suppresses the growth of ESCC cells in a time and dose-dependent manner.

As a whole, our study suggested that dasabuvir is a novel ROCK1 inhibitor and can suppress ESCC in vivo and in vitro (Fig. 7). The clinical safety dosage offers an opportunity for ESCC treatment or recurrence chemoprevention clinical trial.

## Conclusions

Dasabuvir inhibited the phosphorylation of ERK1/2 and downregulated CDK4 and cyclin D1 through targeting ROCK1, thus blocking the progression of ESCC. These results could be benefit for both the future research and clinical use of dasabuvir.

## Supplementary information

supplemental material

original western blots

aj-checklist

## Data Availability

The proteomics data is available online through the ProteomeXchange Consortium via http://proteomecentral.proteomexchange.org/cgi/GetDataset?ID=PXD031186 with the data set identifiers PXD031186.
