# Supplementary material for: Dasabuvir suppresses esophageal squamous cell carcinoma growth in vitro and in vivo through targeting ROCK1
Source: Cell Death Dis. 2023 Feb 13;14(2):118. doi: 10.1038/s41419-023-05633-2 (PMC9924867; doi:10.1038/s41419-023-05633-2)
Supplement: Supplementary file 1 — supplemental material [file 41419_2023_5633_MOESM1_ESM.docx]

Dasabuvir suppresses esophageal squamous cell carcinoma growth *in vitro* and *in vivo* through targeting ROCK1

Xinning Liu^1, 2, 3†^, Yanan Jiang^1, 2. 4, 5†^, Hao Zhou^1, 2†^, Xiaokun Zhao^1, 2^, Mingzhu Li^1, 2^, Zhuo Bao^1, 2^, Zitong Wang^1^, Chenyang Zhang^1^, Zhenliang Xie^1^, Jimin Zhao^1, 4, 5, 7^, Zigang Dong^1,2^, Kangdong Liu^1, 2, 4, 5, 6, 7*^ and Zhiping Guo^1, 2, 8*^

^1^Department of Pathophysiology, School of Basic Medical Sciences, Zhengzhou University, Zhengzhou, Henan, China

^2^China-US Hormel (Henan) Cancer Institute, Zhengzhou, Henan, China

^3^Department of Pulmonary and Critical Care Medicine, Huashan Hospital, Fudan University, Shanghai, China

^4^State Key Laboratory of Esophageal Cancer Prevention and Treatment, Zhengzhou University, Zhengzhou, China

^5^Henan Provincial Cooperative Innovation Center for Cancer Chemoprevention, Zhengzhou, China

^6^Cancer Chemoprevention International Collaboration Laboratory, Zhengzhou, China

^7^Research Center of Basic Medicine, Academy of Medical Sciences, Zhengzhou University

^8^Fuwai Central China Cardiovascular Hospital, Zhengzhou University, Zhengzhou, China

^#^These authors contributed equally to this work.

*Correspondence: Zhiping Guo (zhiping74@hotmail.com)

Kangdong Liu (kdliu@zzu.edu.cn)


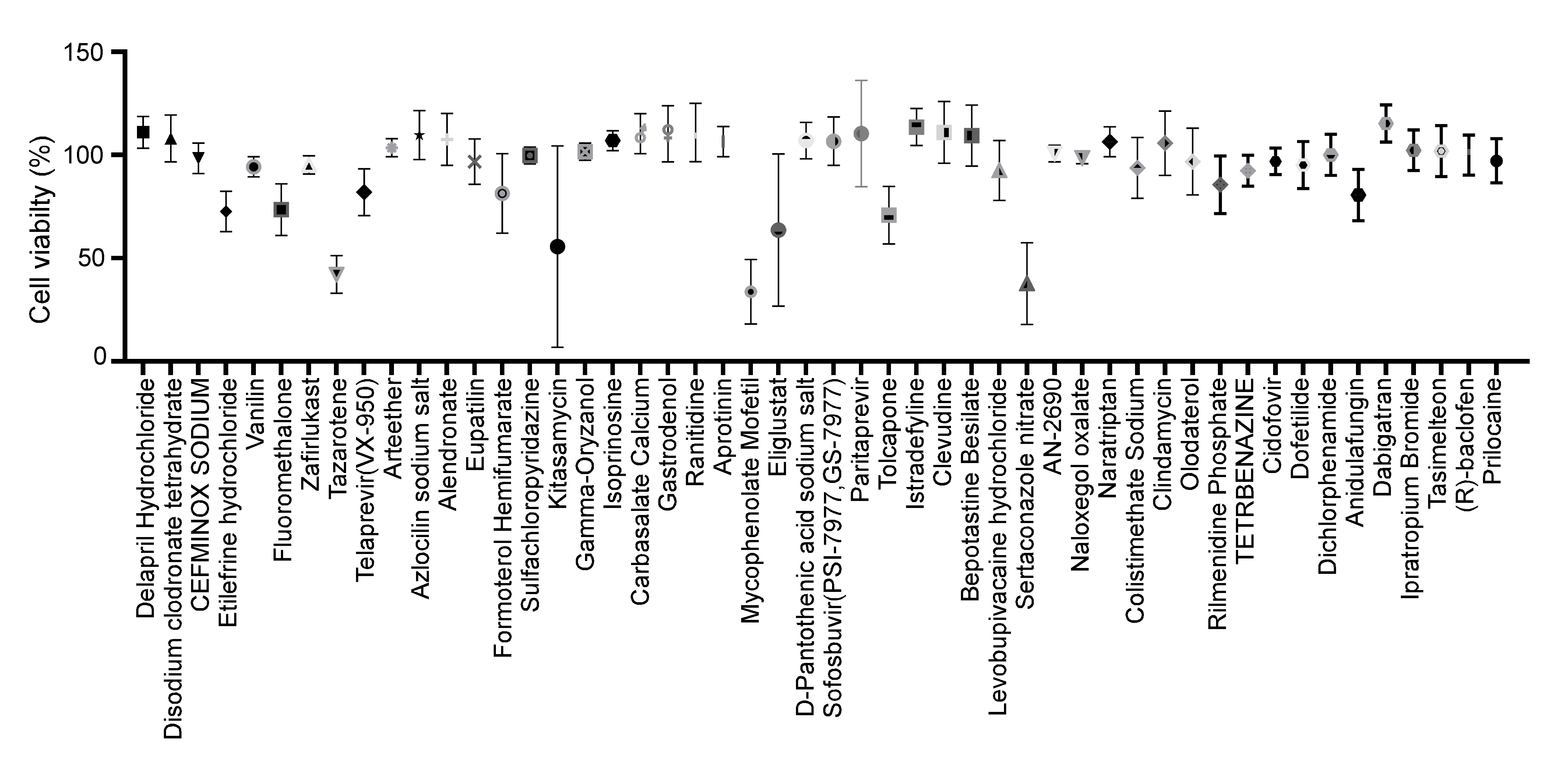


**Figure S1. The cytotoxicity results for KYSE450 treated with 50 different drugs (*n* = 3).**

**
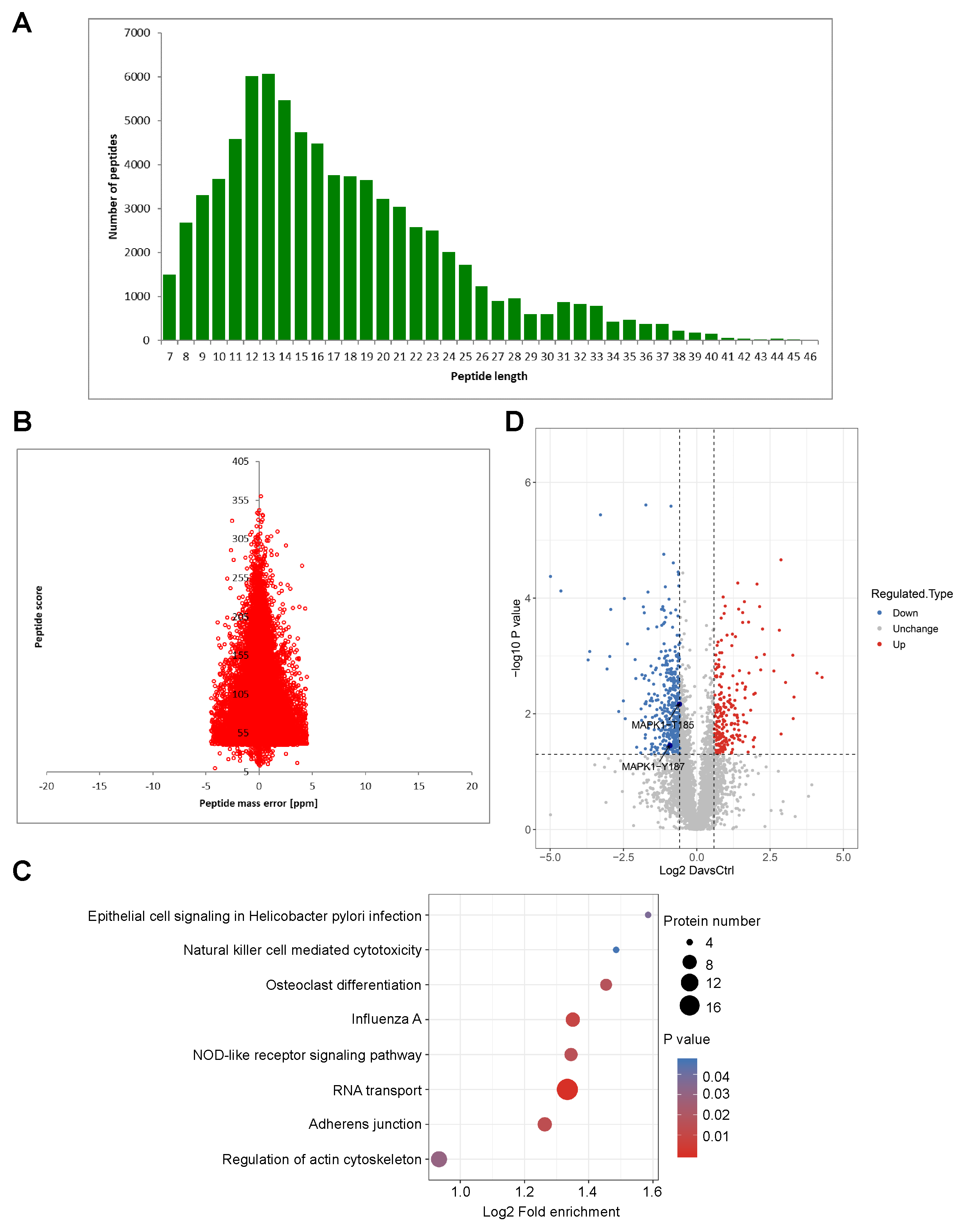
**

**Figure S2. Phosphoproteomics showes dasabuvir downregulates MAPK1 T185, Y187 sites.** (A) Most of the peptides identified by mass spectrometry were distributed in the range of 7-20 amino acids and met the requirements of quality control in phosphoproteomics. (B) The mass accuracy distribution diagram of mass spectrum data showed that the first-order mass error of most of the spectra was within 10 ppm, which was in line with the high precision characteristics of mass spectrum in phosphoproteomics. (C) Bubble chart of KEGG pathways for proteins where altered phosphorylation sites located. (D) Volcano diagram showed MAPK1 T185, Y187 were downregulated.


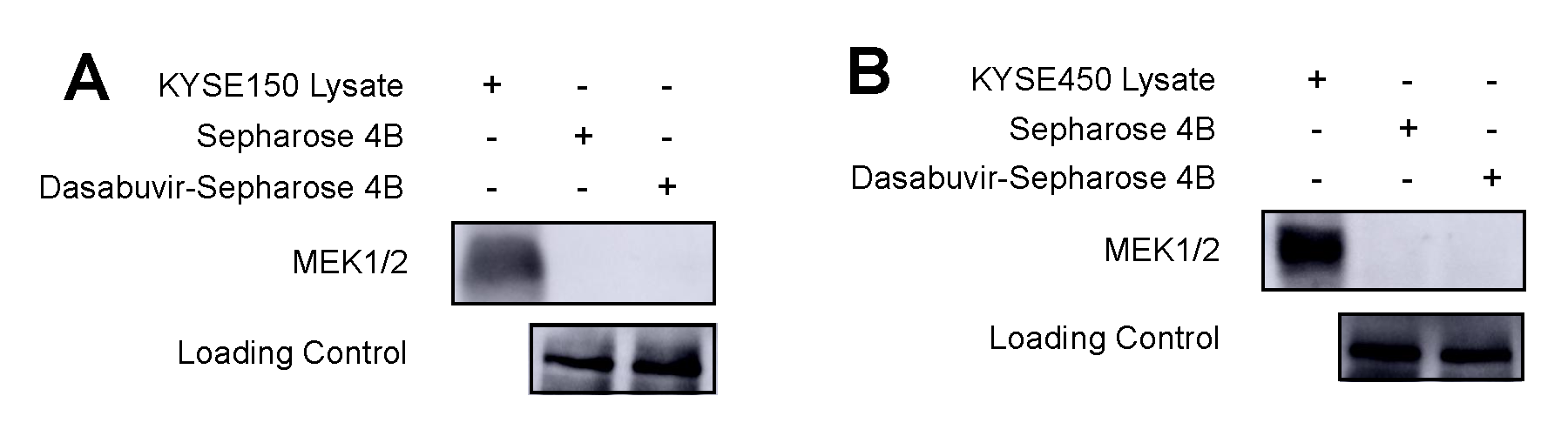


**Figure S3. Dasabuvir can not bind to MEK1/2.** (A) Pulldown assay indicated dasabuvir can not bind to MEK1/2 in KYSE150. (B) Pulldown assay indicated dasabuvir can not bind to MEK1/2 in KYSE450.


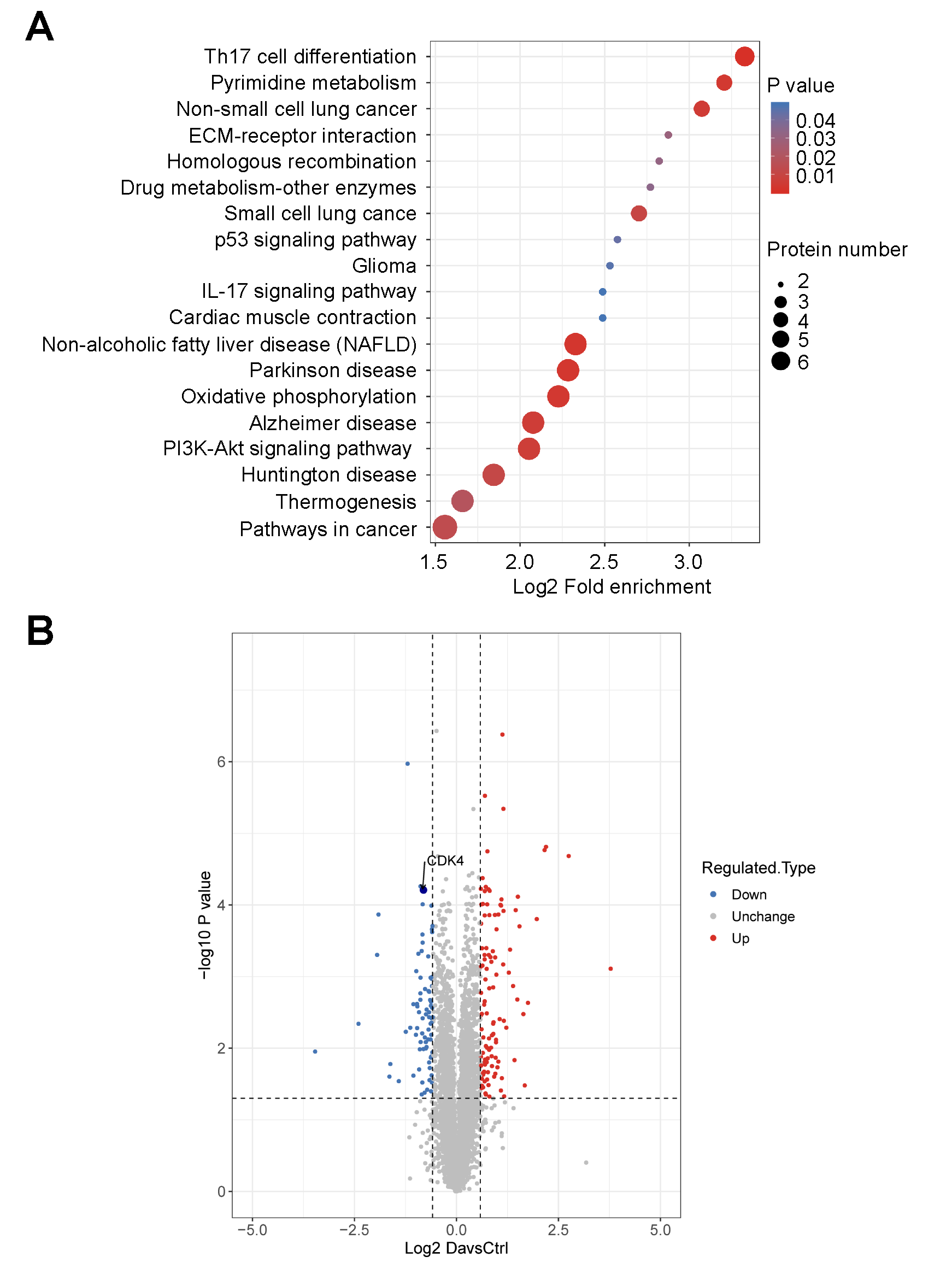


**Figure S4. CDK4 is enriched from proteomics.** (A) Bubble chart of enrichment KEGG pathway analysis of the downregulated proteins. (B) Volcano diagram of showed CDK4 was downregulated.
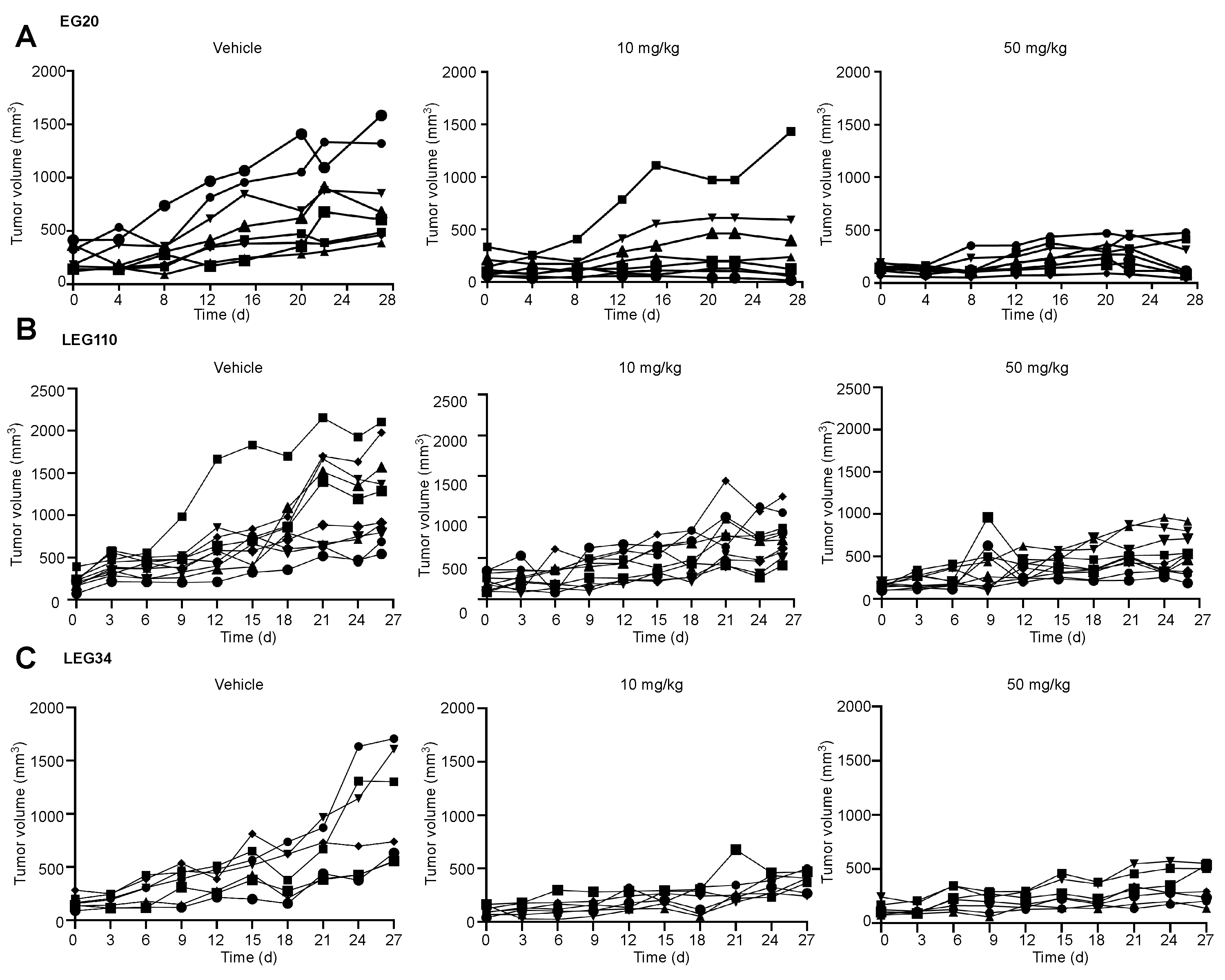


**Figure S5.** **Tumor growth curves for each mouse.** (A) A graph showed the tumor growth curves for each mouse in EG20 (n = 8). (B) A graph showed the tumor growth curves for each mouse in LEG110 (n = 10). (C) A graph showed the tumor growth curves for each mouse in LEG34 (n = 7).

**
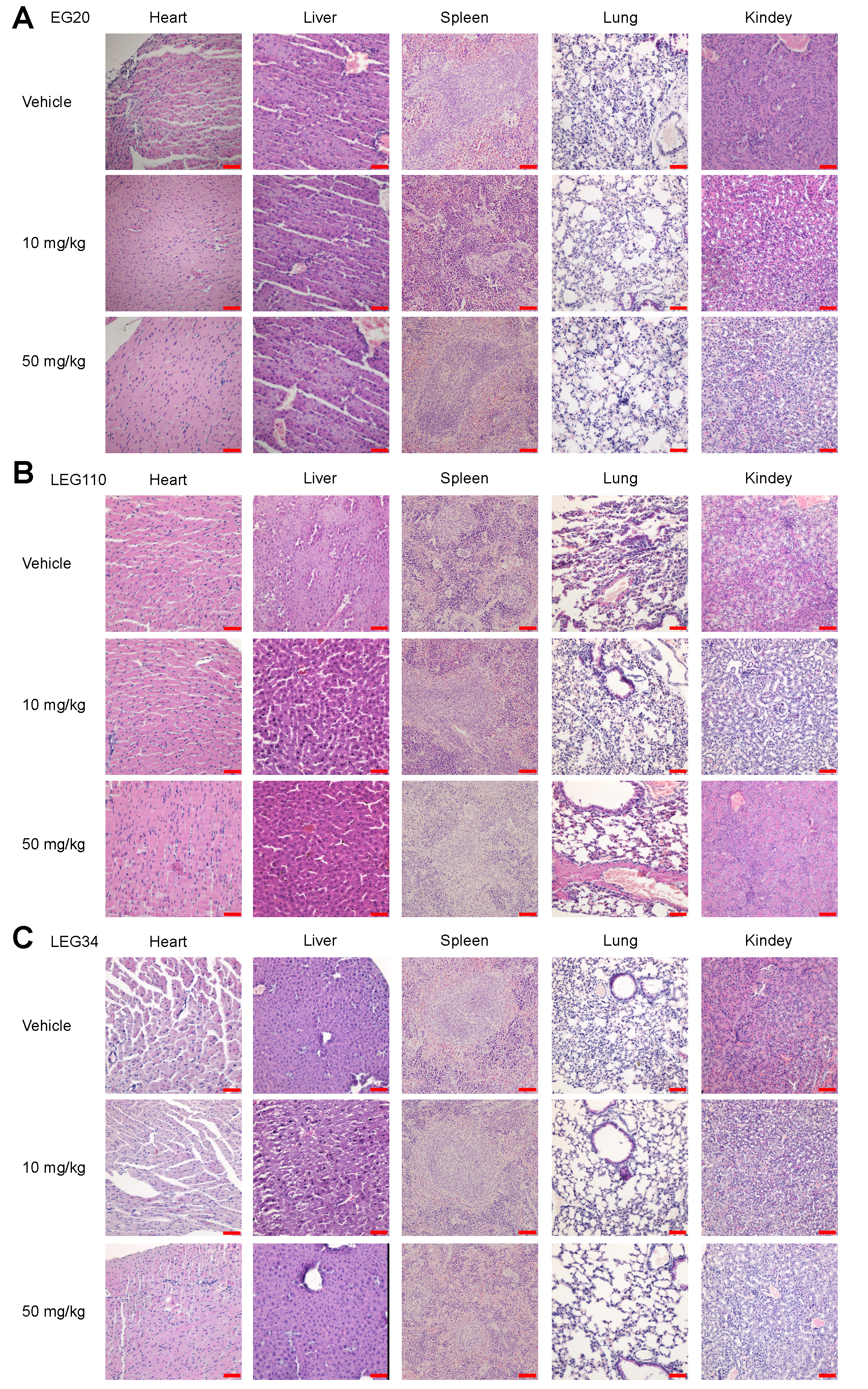
**

**Figure S6.** **HE staining of mice organ tissues.** (A) Representative images of HE staining of heart, liver, spleen, lung and brain from mice for EG20. (B) Representative images of HE staining of heart, liver, spleen, lung and brain from mice for LEG110. (C) Representative images of HE staining of heart, liver, spleen, lung and brain from mice for LEG34. Scale bar = 50 μm.

**Table S1.** **Primer sequences for mutated ROCK1 for RT-qPCR designed by SnapGene.**

| ***Gene*** | **Forward（5'-3'）** | **Reverse（5'-3'）** |
| --- | --- | --- |
| *ROCK1*  *(Met156)* | TCTACATGGTGATGGAATACGCCCCTGGTGGAGATCTTGTAAA | TTTACAAGATCTCCACCAGGGGCGTATTCCATCACCATGTAGA |
| *ROCK1*  *(*Leu202*)* | TTCACAGAGATGTGAAGCCTGCCAACATGCTGCTGGATAAATC | GATTTATCCAGCAGCATGTTGGCAGGCTTCACATCTCTGTGAA |
| *ROCK1*  *(*Asp205*)* | ATGTGAAGCCTGATAACATGGCCCTGGATAAATCTGGACATTT | AAATGTCCAGATTTATCCAGGGCCATGTTATCAGGCTTCACAT |

**Table S2.** **The shRNA sequences for ROCK1.**

| ***shROCK1*** | **Forward（5'-3'）** | **Reverse（5'-3'）** |
| --- | --- | --- |
| #1 | CCGGGCCAGCAAAGAGAGTGATATTCTCGAGAATATCACTCTCTTTGCTGGCTTTTTG | AATTCAAAAAGCCAGCAAAGAGAGTGATATTCTCGAGAATATCACTCTCTTTGCTGGC |
| #2 | CCGGGTGGAGATCTTGTAAACTTAACTCGAGTTAAGTTTACAAGATCTCCACTTTTTG | AATTCAAAAAGTGGAGATCTTGTAAACTTAACTCGAGTTAAGTTTACAAGATCTCCAC |
| #3 | CCGGGCATTCCAAGATGATCGTTATCTCGAGATAACGATCATCTTGGAATGCTTTTTG | AATTCAAAAAGCATTCCAAGATGATCGTTATCTCGAGATAACGATCATCTTGGAATGC |
| #4 | CCGGCGGTTAGAACAAGAGGTAAATCTCGAGATTTACCTCTTGTTCTAACCGTTTTTG | AATTCAAAAACGGTTAGAACAAGAGGTAAATCTCGAGATTTACCTCTTGTTCTAACCG |
| #5 | CCGGGCACCAGTTGTACCCGATTTACTCGAGTAAATCGGGTACAACTGGTGCTTTTTG | AATTCAAAAAGCACCAGTTGTACCCGATTTACTCGAGTAAATCGGGTACAACTGGTGC |

**Table S3. SwissTargetPrediction results showed the possible targets of dasabuvir.**

**SwissTargetPrediction**

| **Target** | **Common name** | **Uniprot ID** | **ChEMBL ID** | **Target Class** | **Probability*** | **Known actives (3D/2D)** |
| --- | --- | --- | --- | --- | --- | --- |
| Rho-associated protein kinase 1 | ROCK1 | Q13464 | CHEMBL3231 | Kinase | 0.110612204 | 108 / 0 |
| p53-binding protein Mdm-2 | MDM2 | Q00987 | CHEMBL5023 | Other nuclear protein | 0.110612204 | 193 / 0 |
| Cystic fibrosis transmembrane conductance regulator | CFTR | P13569 | CHEMBL4051 | Other ion channel | 0.110612204 | 15 / 0 |
| Vasopressin V1b receptor | AVPR1B | P47901 | CHEMBL1921 | Family A G protein-coupled receptor | 0.110612204 | 30 / 0 |
| Platelet activating factor receptor | PTAFR | P25105 | CHEMBL250 | Family A G protein-coupled receptor | 0.110612204 | 71 / 0 |
| Cathepsin K | CTSK | P43235 | CHEMBL268 | Protease | 0.110612204 | 112 / 0 |
| Cathepsin S | CTSS | P25774 | CHEMBL2954 | Protease | 0.110612204 | 117 / 0 |
| Cathepsin L | CTSL | P07711 | CHEMBL3837 | Protease | 0.110612204 | 101 / 0 |
| Calcitonin gene-related peptide type 1 receptor | CALCRL | Q16602 | CHEMBL3798 | Family B G protein-coupled receptor | 0.110612204 | 251 / 0 |
| Protein Mdm4 | MDM4 | O15151 | CHEMBL1255126 | Unclassified protein | 0.110612204 | 19 / 0 |
| Proteinase-activated receptor 1 | F2R | P25116 | CHEMBL3974 | Family A G protein-coupled receptor | 0.110612204 | 60 / 0 |
| Bile acid receptor FXR | NR1H4 | Q96RI1 | CHEMBL2047 | Nuclear receptor | 0.110612204 | 24 / 0 |
| Thrombin | F2 | P00734 | CHEMBL204 | Protease | 0.110612204 | 289 / 0 |
| Trypsin I | PRSS1 | P07477 | CHEMBL209 | Protease | 0.110612204 | 29 / 0 |
| Urokinase-type plasminogen activator | PLAU | P00749 | CHEMBL3286 | Protease | 0.110612204 | 22 / 0 |
| Carnitine O-palmitoyltransferase 1, liver isoform | CPT1A | P50416 | CHEMBL1293194 | Enzyme | 0.110612204 | 75 / 0 |
| Thyroid stimulating hormone receptor | TSHR | P16473 | CHEMBL1963 | Family A G protein-coupled receptor | 0.110612204 | 18 / 0 |
| 6-phosphofructo-2-kinase/fructose-2,6-bisphosphatase 3 | PFKFB3 | Q16875 | CHEMBL2331053 | Enzyme | 0.110612204 | 178 / 0 |
| TGF-beta receptor type I | TGFBR1 | P36897 | CHEMBL4439 | Kinase | 0.110612204 | 117 / 0 |
| Tissue-type plasminogen activator | PLAT | P00750 | CHEMBL1873 | Protease | 0.110612204 | 13 / 0 |
