# Supplementary material for: Dasabuvir suppresses esophageal squamous cell carcinoma growth in vitro and in vivo through targeting ROCK1
Source: Cell Death Dis. 2023 Feb 13;14(2):118. doi: 10.1038/s41419-023-05633-2 (PMC9924867; doi:10.1038/s41419-023-05633-2)
Supplement: Supplementary file 2 — original western blots [file 41419_2023_5633_MOESM2_ESM.docx]

**The whole uncropped images of the original western blots in Fig. 2E.**


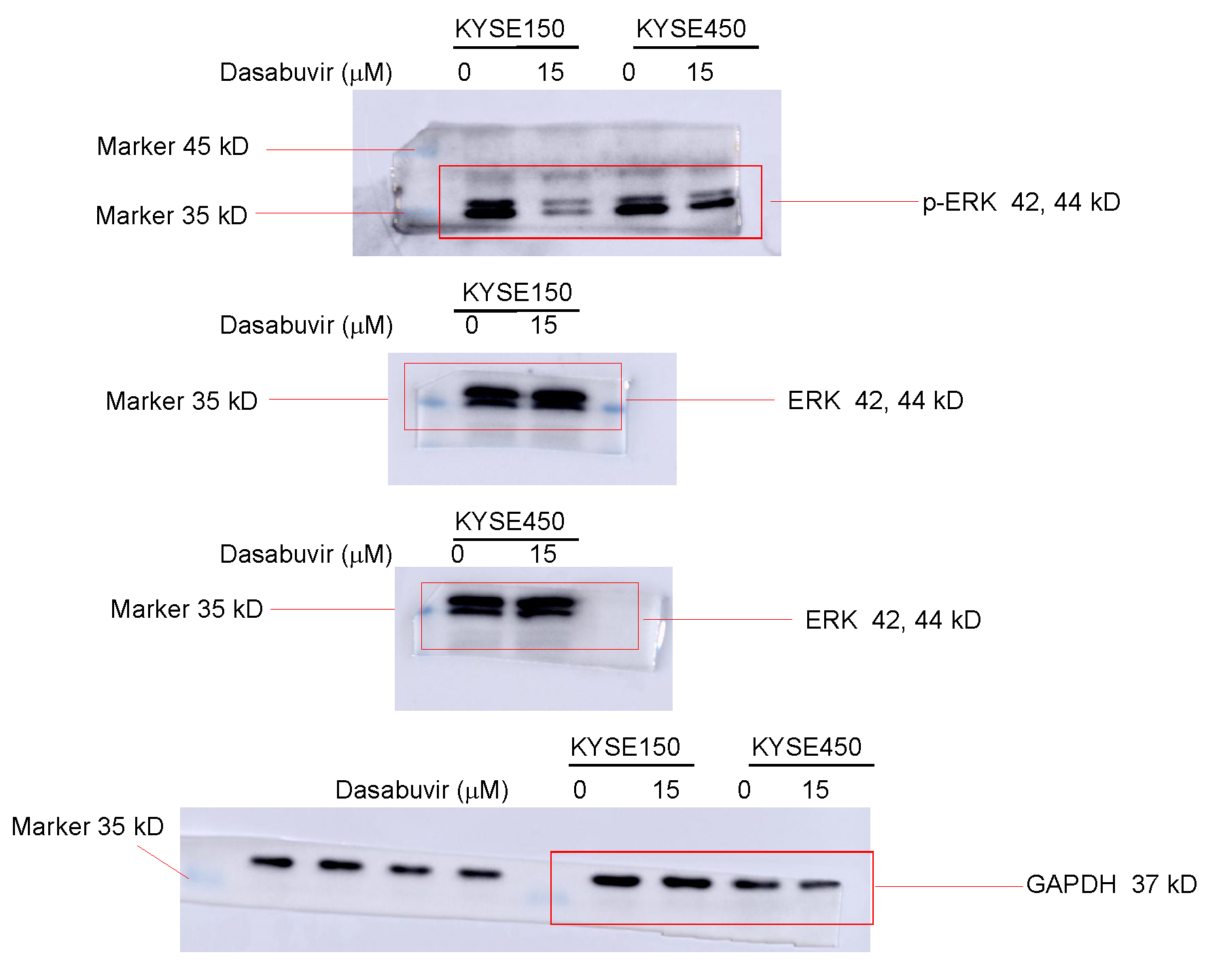


**The whole uncropped images of the original western blots in Fig. 3.**

**
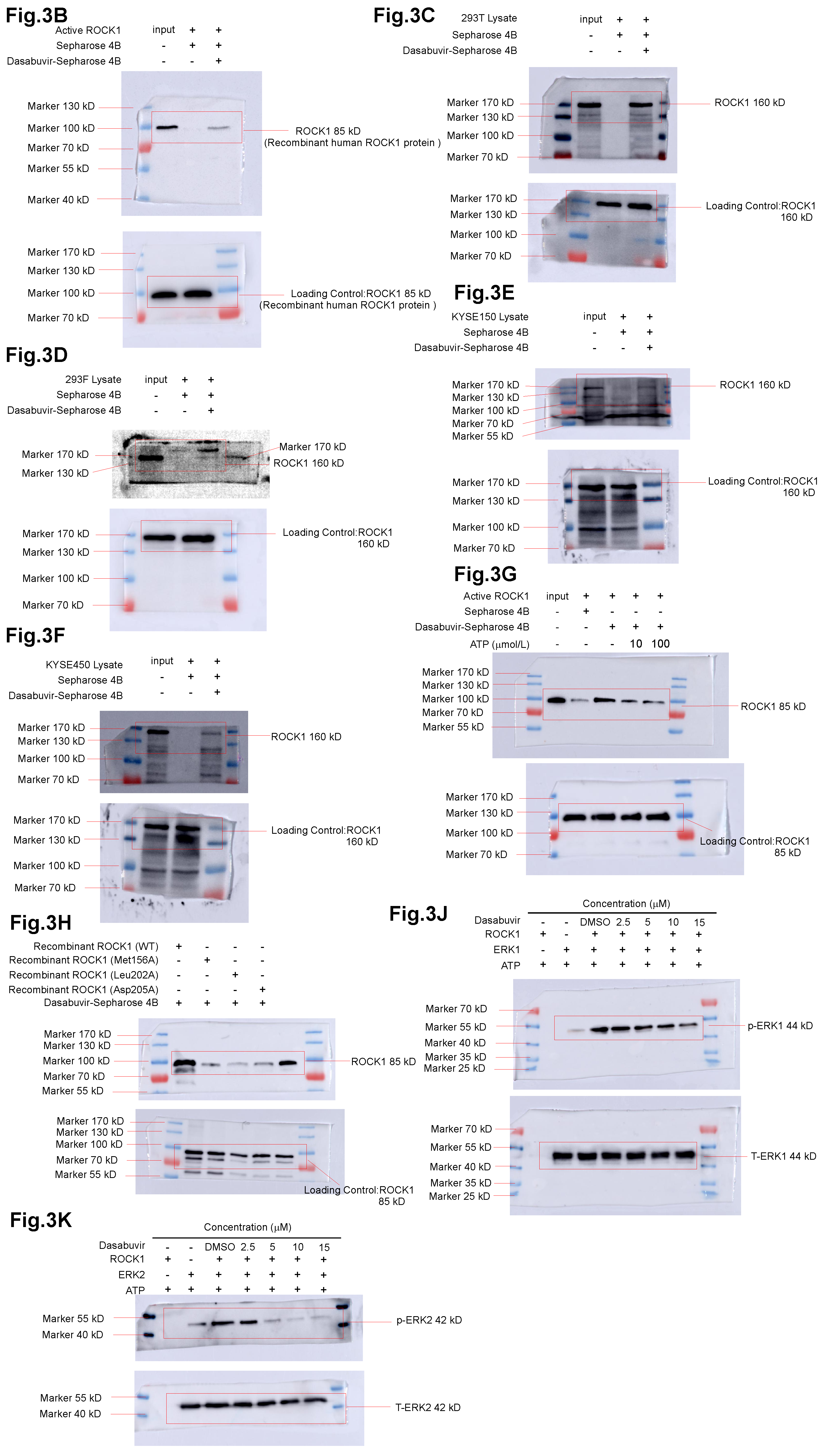
**

**The whole uncropped images of the original western blots in Fig. 4E.**


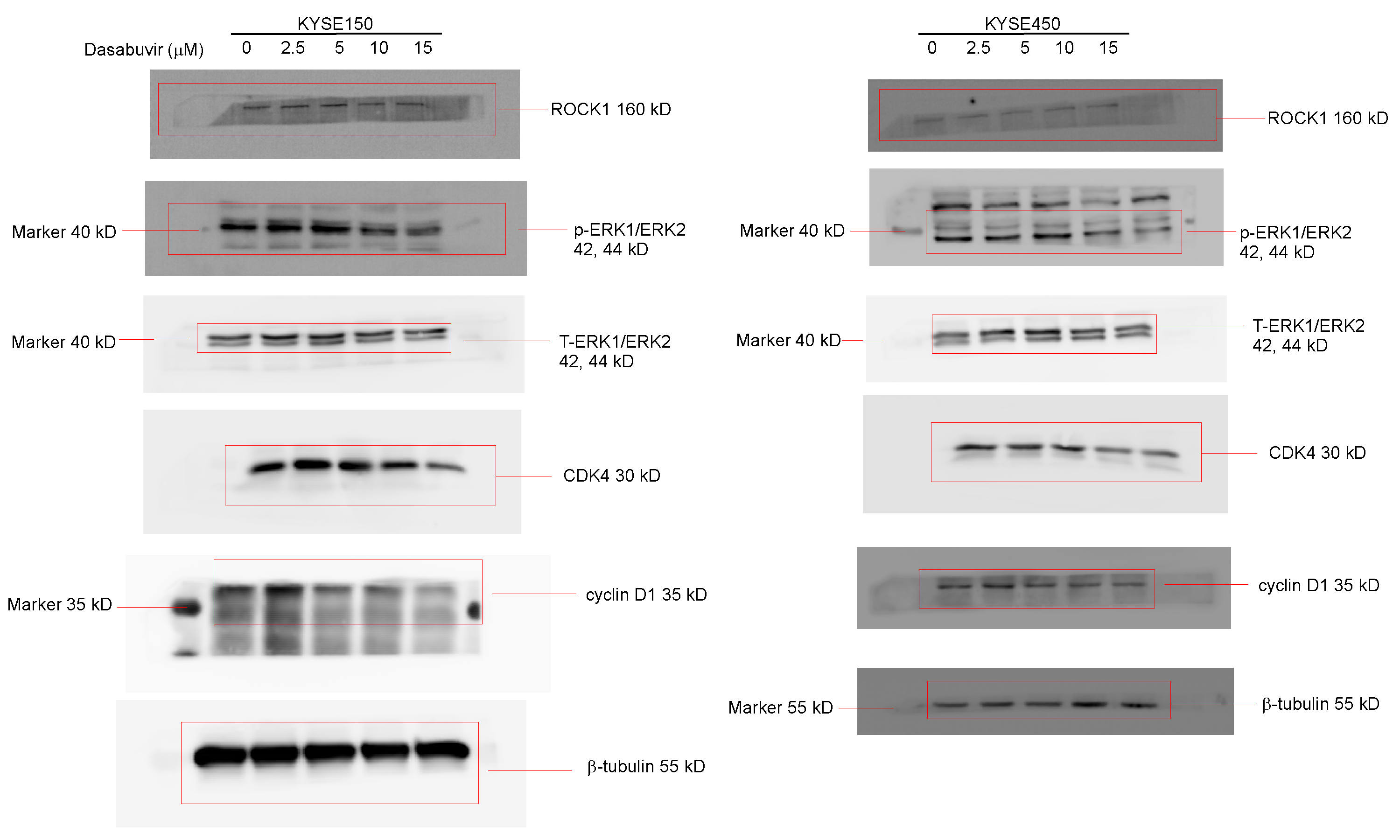


**The whole uncropped images of the original western blots in Fig. 5.**

**
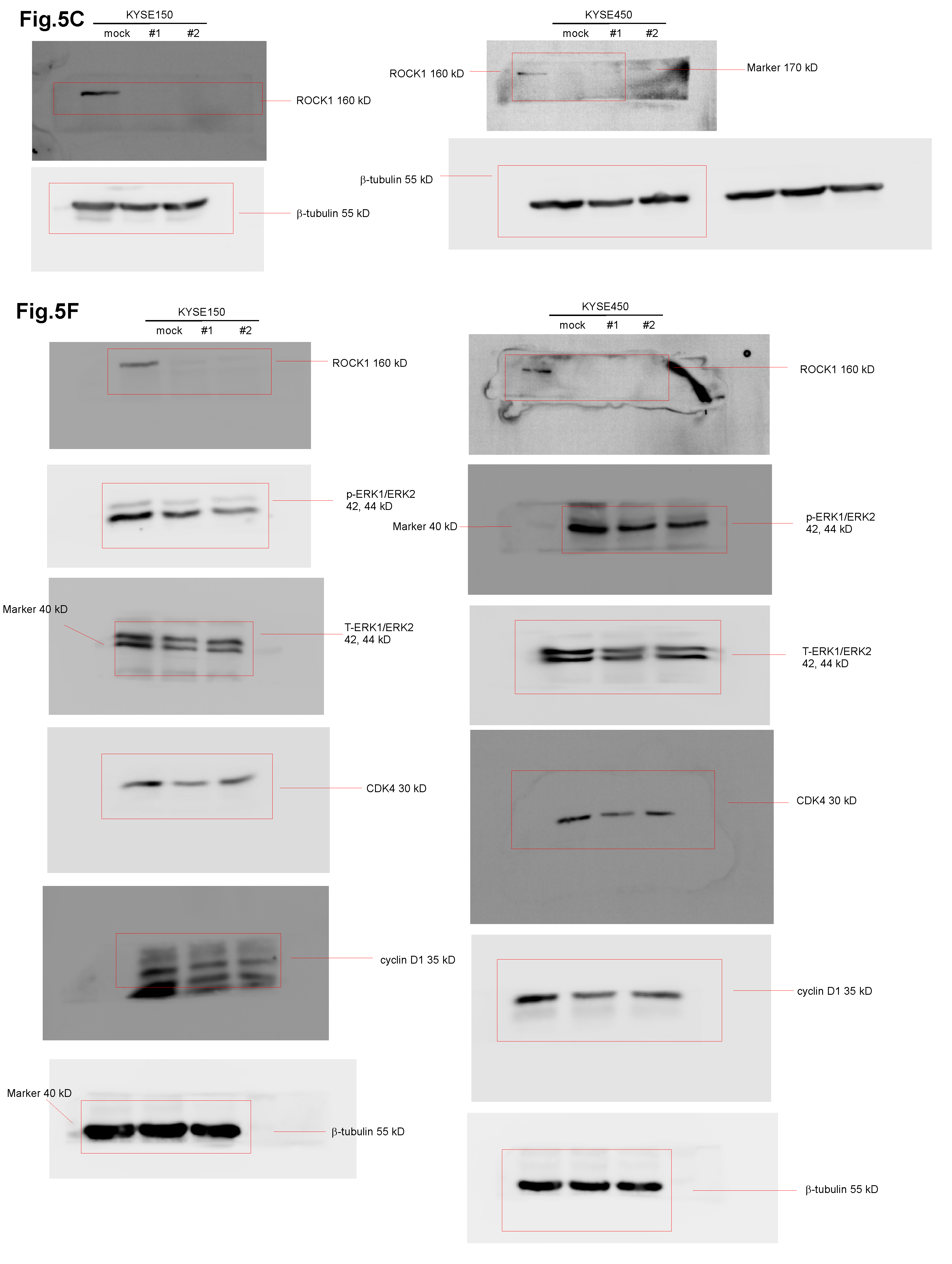
**

**The whole uncropped images of the original western blots in Fig. S2.**

**
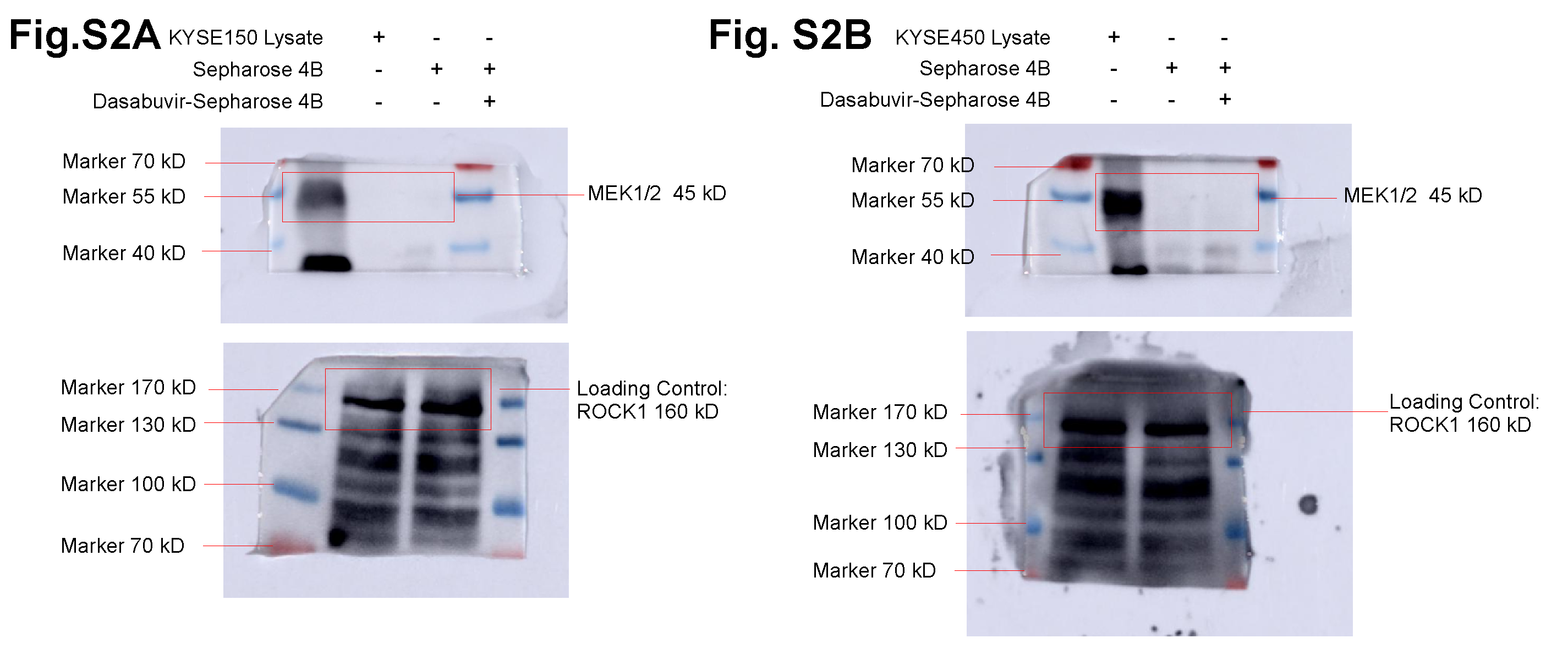
**
